# *Ascl1 *is a required downstream effector of *Gsx *gene function in the embryonic mouse telencephalon

**DOI:** 10.1186/1749-8104-4-5

**Published:** 2009-02-10

**Authors:** Bei Wang, Ronald R Waclaw, Zegary J Allen, Francois Guillemot, Kenneth Campbell

**Affiliations:** 1 Division of Developmental Biology, Cincinnati Children's Hospital Medical Center, University of Cincinnati College of Medicine, 3333 Burnet Avenue, Cincinnati OH45229, USA; 2 Division of Molecular Neurobiology, NIMR, The Ridgeway, Mill Hill, London, UK; 3 Nina Ireland Laboratory of Developmental Neurobiology, Center for Neurobiology and Psychiatry, Rock Hall, UCSF 1550 4th Street, San Francisco, CA 94158, USA

## Abstract

**Background:**

The homeobox gene *Gsx2 *(formerly *Gsh2*) is known to regulate patterning in the lateral ganglionic eminence (LGE) of the embryonic telencephalon. In its absence, the closely related gene *Gsx1 *(previously known as *Gsh1*) can partially compensate in the patterning and differentiation of ventral telencephalic structures, such as the striatum. However, the cellular and molecular mechanisms underlying this compensation remain unclear.

**Results:**

We show here that in the *Gsx2 *mutants Gsx1 is expressed in only a subset of the ventral telencephalic progenitors that normally express Gsx2. Based on the similarities in the expression of Gsx1 and Ascl1 (Mash1) within the *Gsx2 *mutant LGE, we examined whether Ascl1 plays an integral part in the *Gsx1*-based recovery. *Ascl1 *mutants show only modest alterations in striatal development; however, in *Gsx2;Ascl1 *double mutants, striatal development is severely affected, similar to that seen in the *Gsx1;Gsx2 *double mutants. This is despite the fact that *Gsx1 *is expressed, and even expands, in the *Gsx2;Ascl1 *mutant LGE, comparable to that seen in the *Gsx2 *mutant. Finally, Notch signaling has recently been suggested to be required for normal striatal development. In spite of the fact that Notch signaling is severely disrupted in *Ascl1 *mutants, it actually appears to be improved in the *Gsx2;Ascl1 *double mutants.

**Conclusion:**

These results, therefore, reveal a non-proneural requirement of *Ascl1 *that together with *Gsx1 *compensates for the loss of *Gsx2 *in a subset of LGE progenitors.

## Background

The homeobox gene *Gsx2 *(formerly known as *Gsh2*) has been shown to be required for correct dorsal-ventral patterning in the embryonic mouse telencephalon [1-3]. *Gsx2 *accomplishes this by repressing dorsal telencephalic genes such as *Pax6 *and promoting the expression of ventral regulators such as *Ascl1 *(*Mash1*) and *Dlx *genes within ventricular zone (VZ) and subventricular zone (SVZ) progenitors of the lateral ganglionic eminence (LGE). Although *Gsx2 *mutants do not survive after birth [4], analyses at late embryonic stages have demonstrated severe reductions in markers of striatal projection neurons as well as olfactory bulb interneurons [1-3,5,6], both of which are derived from the LGE [7-10].

The closely related *Gsx1 *(*Gsh1*) is also expressed in the embryonic ventral telencephalon [11], although no telencephalic phenotype has been reported [5,6]. Despite this, removal of *Gsx1 *on the *Gsx2 *mutant background eliminates nearly all striatal projection neurons and olfactory bulb interneurons, suggesting that *Gsx1 *can, at least in part, compensate for the loss of *Gsx2 *in the development of these ventral telencephalic structures. This compensation, however, is complex because *Gsx1 *is normally only present in the medial ganglionic eminence and the ventral-most portion of the LGE. In *Gsx2 *mutants, *Gsx1 *expression spreads dorsally to encompass the mutant LGE at mid-neurogenesis time points (for example, embryonic day (E)14), which is coincident with the re-establishment of ventral identity (for example, *Ascl1 *and *Dlx *expression) in the mutant LGE [5,6]. Both *Ascl1 *and *Dlx *genes are known to be required for normal development of the striatum and olfactory bulb interneurons [12-16]. Moreover, a recent study [17] suggests that *Ascl1 *and *Dlx *genes control distinct and parallel pathways that act in the specification of olfactory bulb interneurons. The mechanism by which *Gsx1 *compensates for the loss of *Gsx2 *has not been fully elucidated. Moreover, the requirement for *Ascl1 *or *Dlx *genes in this process is unclear.

In this study we have examined the molecular mechanisms underlying *Gsx1*-mediated recovery of ventral telencephalic development in *Gsx2 *mutants. To do this, we have generated and analyzed *Gsx2*^*EGFP *^mice as well as *Gsx2;Ascl1 *double mutants at multiple embryonic stages. Removal of *Ascl1 *from the *Gsx2 *mutant background results in a telencephalic phenotype nearly identical to the *Gsx1:Gsx2 *double mutant [5,6]. These results thus indicate that *Ascl1 *is an essential component of the *Gsx1*-mediated recovery in a subset of LGE progenitors within the *Gsx2 *mutant telencephalon.

## Results

### Gsx1 expression in the *Gsx2 *mutant telencephalon

**Figure 1:**
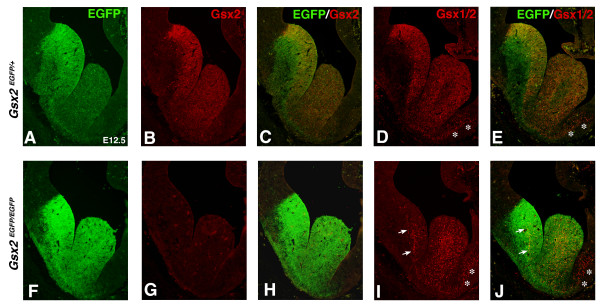
**Knock-in of enhanced green fluorescent protein (EGFP) into the *Gsx2 *locus**. (A, B) EGFP expression in Gsx2^*EGFP*/+ ^embryos (A) recapitulates endogenous Gsx2 expression at E12.5 (B). **(C) **Note that EGFP expression persists longer than Gsx2 protein expression in the lateral ganglionic eminence (LGE; merged image). – (D) The Gsx1/2 antibody detects expression of Gsx1 and Gsx2 in the ventral telencephalon. (E) Note that the EGFP expression is absent in the septal expression domain of the Gsx1/2 antibody (asterisks in D, E, I, J) indicating Gsx1-specific expression. **(F-H) **Homozygous knock-in of EGFP into the Gsx2 locus (*Gsx2*^*EGFP*/*EGFP*^) (F) results in a loss of function of Gsx2 with no detectable protein expression (G). Note that the EGFP expression is more intense in the *Gsx2*^*EGFP*/*EGFP *^embryos (H) compared to the *Gsx2*^*EGFP*/+ ^embryos (A). **(I) **The initial expansion of Gsx1 into the LGE of *Gsx2*^*EGFP*/*EGFP*^embryos is detectable at E12.5 with an anti-Gsx1/2 antibody. **(J) **Gsx1 expression is not observed in all Gsx2 mutant cells of the LGE (merged image) but found largely at the VZ/SVZ boundary (arrows in I, J).

Previous studies have shown that *Gsx1 *expands in the LGE of the *Gsx2 *mutant [3,5,6]. Normally, the cells expressing *Gsx1 *are confined to the ventral-most portion of the LGE; however, in *Gsx2 *mutants the expression of this gene expands throughout the entire dorsal-ventral extent of the LGE between E11 and E14. We have generated mice in which the first exon of *Gsx2 *is interrupted by an IRES-enhanced green fluorescent protein (EGFP) cassette so that EGFP is expressed in place of Gsx2. These mice appear to faithfully reproduce Gsx2 expression and provide a short-term fate map of Gsx2 derived cells that no longer express the protein (Figure 1A–C). Moreover, the *Gsx2*^*EGFP*/*EGFP *^embryos lack Gsx2 protein expression, while still delineating the portion of the LGE that the targeted *Gsx2 *gene is being transcribed in by virtue of the EGFP staining (Figure 1F–H). These mutants exhibit identical patterning defects to those reported for the previously available *Gsx2 *mutant allele [1-4] (data not shown). Using these mice together with an antibody that detects both Gsx1 and Gsx2 [18], we were able to examine the Gsx1 recovery on a cellular level, within the context of the Gsx2 expression domain.

**Figure 2:**
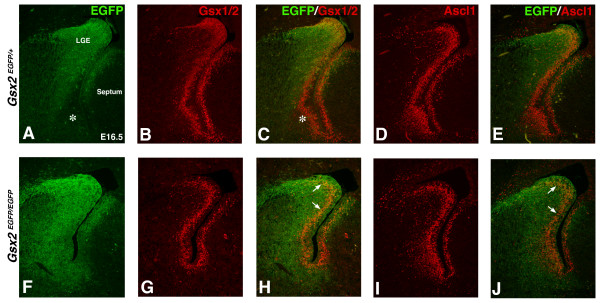
**Expansion of Gsx1 in the *Gsx2*^*EGFP*/*EGFP *^lateral ganglionic eminence (LGE) occurs in only a subset of cells at the ventricular zone (VZ)/subventricular zone (SVZ) border**. **(A-C) **Control embryos (*Gsx2*^*EGFP*/+^) express Gsx2 and enhanced green fluorescent protein (EGFP) throughout the VZ of the LGE at E16.5. In addition, the Gsx1/2 antibody labels scattered cells in the SVZ (B) where EGFP expression is observed in the majority of the SVZ (A, C). Asterisks in (A, C) mark Gsx1/2 staining in the remnant of the medial ganglionic eminence. Because EGFP expression from the Gsx2 locus is not found in this region, it is likely that the staining reflects Gsx1 expression. **(G) **The expansion of Gsx1 in *Gsx2*^*EGFP*/*EGFP *^embryos is throughout the LGE at E16.5. **(F, H) **Note that Gsx1 expression in *Gsx2*^*EGFP*/*EGFP *^embryos is observed only at the VZ/SVZ border in the LGE (arrows in merged image in (H)) whereas EGFP expression (labeling Gsx2 mutant cells) is observed throughout the VZ and the SVZ (F, H). **(D, E) **Control embryos (*Gsx2*^*EGFP*/+^) express Ascl1 at highest levels near the VZ/SVZ border (D) and only in scattered cells of the VZ (merged image with EGFP in (E)). **(I) **By E16.5, Ascl1 is recovered in the LGE of *Gsx2*^*EGFP*/*EGFP *^embryos predominately at the VZ/SVZ border (arrows in (J)), which is similar to the expansion of Gsx1 expression in these mutants (G).

While the Gsx1/2 antibody staining looks very similar to that of Gsx2 in the *Gsx2*^*EGFP*/+ ^embryos (Figure 1A–E), it reveals a rather different pattern in the *Gsx2*^*EGFP*/*EGFP *^embryos (Figure 1I, J). At E12.5, the cells expressing Gsx1 in the mutant LGE are few in number and largely confined to its ventral half. This finding is in agreement with previous *Gsx1 *gene expression studies [3,5]; however, the cellular resolution afforded by the immunohistochemical approach revealed that the Gsx1 cells appear mostly at the border between the VZ and SVZ (Figure 1I, J). This is different from Gsx2 expression in the wild-type LGE where cells throughout the apical-basal extent of the VZ express this protein, albeit at different levels of expression (Figure 1B). Previous studies have shown that the expansion of *Gsx1 *throughout the *Gsx2 *mutant LGE is complete between E14.5 and E16.5 [3,5,6]. This is clearly revealed by Gsx1/2 staining in the *Gsx2*^*EGFP*/*EGFP *^mutants at E16.5 (Figure 2G). At this stage, only around half the LGE cells that would normally express Gsx2 contain Gsx1 staining. Again, the majority of the Gsx1 expressing cells appear to line up at the VZ/SVZ boundary (Figure 2H). This is similar to Gsx1/2 staining in the remnant of the medial ganglionic eminence in *Gsx2*^*EGFP*/+ ^brains (Figure 2B), and since this staining does not coincide with the EGFP from the Gsx2 locus (Figure 2A–C), it is likely to reflect Gsx1 expression in the wild-type medial ganglionic eminence. Thus, although Gsx1 can at least partially compensate for Gsx2 [5,6], it does not do so in all cells of the LGE that would normally express Gsx2 but only in a subpopulation positioned at the VZ/SVZ boundary.

### Relationship between Gsx1 and Ascl1 in the *Gsx2 *mutant LGE

Ascl1 (Mash1) is known to be required for the normal development of the ventral telencephalon [14-16]. Furthermore, Ascl1 is dependent on Gsx2 for its normal expression in LGE progenitor cells [1-3], at least at early stages, before Gsx1 expression expands into the mutant LGE. Ascl1 is expressed by many cells within the *Gsx2*^*EGFP*/+ ^VZ, although they are mainly located at the VZ/SVZ boundary (Figure 2D, E). Interestingly, the pattern of Ascl1 expression in the Gsx2 mutants is very similar to that of Gsx1 (as revealed by Gsx1/2 staining; Figure 2G–J), with scattered cells in the VZ and the majority accumulated along the VZ/SVZ boundary. In *Gsx1;Gsx2 *double mutants, Ascl1 is not expressed in the LGE at early stages (for example, E12.5) but at later stages (for example, E15.5–16.5) it is found at low levels within the presumptive LGE region [5,6]. This suggests that although Gsx proteins are not absolutely required for Ascl1 expression in the LGE, they are positive regulators of its expression. The overlap in Gsx1 and Ascl1 expression in the *Gsx2 *mutant LGE (Figure 2H, J) therefore suggests that Ascl1 may act in concert with Gsx1 for the compensation observed in *Gsx2 *mutants.

### Expression of *Gsx2 *in the *Ascl1 *mutant

**Figure 3:**
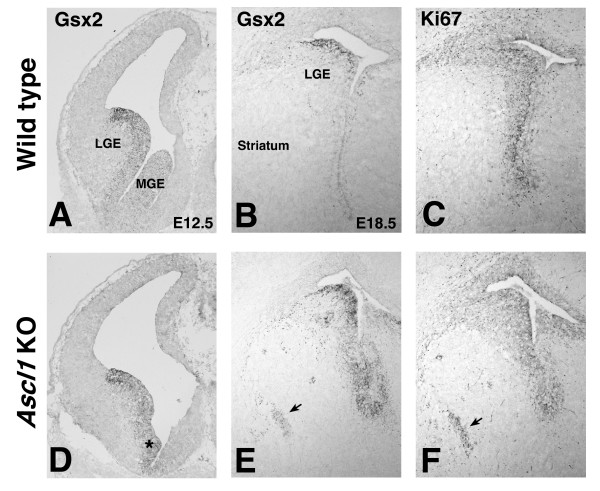
**Increase in progenitor cell markers in the lateral ganglionic eminence (LGE) of Ascl1 mutants**. **(A, D) **At E12.5, Ascl1 mutants express Gsx2 in a relatively normal pattern in the LGE compared to wild type. Note the odd morphology in the developing medial ganglionic eminence (MGE) of Ascl1 mutants (asterisk in (D)). **(B, E) **By E18.5, Gsx2 protein expression is increased in the entire LGE region of Ascl1 mutants (E) compared to controls (B). These ectopic Gsx2 positive cells are observed in the SVZ and striatum, many appearing as clumps of cells stuck in the parenchyma (arrow in (E)). **(C, F) **Dividing cells labeled by Ki67 expression are also increased in the Ascl1 mutant LGE area (F) compared to control (C). Similar to Gsx2, many ectopic Ki67 positive cells appear as clumps (arrow in (F)).

Although Ascl1 expression in LGE progenitors has been shown to require Gsx2 [1-3], the role (if any) of Ascl1 in regulating Gsx2 expression has not been reported. In E12.5 *Ascl1*^-/- ^mutants, we found that Gsx2 (and Gsx1/2) staining in the LGE was not significantly different from that observed in wild types (Figure 3A, D and data not shown). Conversely, at E18.5 we observed a large increase in the numbers of cells expressing Gsx2 along the dorsal-ventral aspect of the VZ in the *Ascl1 *mutants and in certain cases clusters of Gsx2 expressing cells were found in the forming striatum (Figure 3E). The expression of Gsx2 coincided with Ki67 staining in many of these clusters (Figure 3E, F) on closely adjacent sections, suggesting that despite their ectopic location, these Gsx2 cells may remain in the cell cycle. *Gsx1 *expression was not changed in the LGE of *Ascl1 *mutants [19] (data not shown). These findings could indicate that, in addition to being downstream of *Gsx *genes, Ascl1 may also serve a negative feedback function to repress Gsx2 in LGE progenitors, particularly at late embryonic stages.

### *Gsx2;Ascl1 *double mutants exhibit severe striatal defects

**Figure 4:**
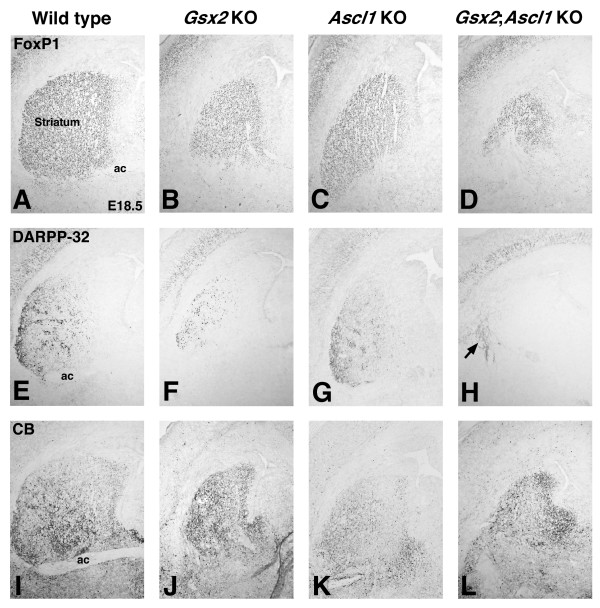
**Removal of *Ascl1 *on the *Gsx2 *mutant background exacerbates the *Gsx2 *mutant phenotype in the striatum**. **(A) **FoxP1 expression labels striatal projection neurons at E18.5. **(B) **In *Gsx2 *mutants, the expression domain of FoxP1 in the striatum is severely reduced. **(D) **Removal of Ascl1 on the Gsx2 mutant background (*Gsx2;Ascl1 *double mutant) results in a more severe effect on the FoxP1 expression domain compared to *Gsx2 *mutants (compare (D) to (B)). **(C) ***Ascl1 *mutants display relatively normal expression of FoxP1 in the striatum. **(E-G) ***Gsx2 *mutants also exhibit a severe reduction in DARPP-32 expression (F), which is enriched in early born striatal neurons at E18.5 in controls (E) and Ascl1 mutants (G). **(H) ***Gsx2;Ascl1 *double mutants display a more severe phenotype in DARPP-32 expression compared to *Gsx2 *mutants (compare (H) to (F)). Note that *Gsx2;Ascl1 *double mutants display a complete loss of DARPP-32 positive neurons in the striatum (H). The only DARPP-32 staining observed in the double mutant striatum is in fibers (arrow in (H)), which presumably arise from the cortical DARPP-32 expressing neurons. **(I, J, L) **Calbindin (CB) expression labels the later born striatal neurons at E18.5 (I) and is upregulated in the SVZ of *Gsx2 *mutants (J) and *Gsx2;Ascl1 *double mutants (L). **(K) ***Ascl1 *mutants exhibit a noticeable reduction in CB expression in the striatum. ac, anterior commissure.

To examine the possibility that Ascl1 is required for the Gsx1-mediated recovery observed in the *Gsx2 *mutant, we generated *Gsx2;Ascl1 *double homozygous mutants and analyzed the striatum at E18.5. The expression of FoxP1 can be used to mark striatal neurons and, thus, the forming striatum at this stage [20,21]. Staining for this marker shows that the size of the striatum in *Gsx2 *mutants is severely reduced compared to wild types (Figure 4A, B), which is consistent with previous studies [1-3,5,6]. Unlike the *Gsx2 *mutants, however, *Ascl1 *mutants exhibit more subtle defects in striatal development [14] and, accordingly, showed a more modest reduction in FoxP1 expression (Figure 4C). Interestingly, the *Gsx2;Ascl1 *double mutants showed an even more severe reduction in FoxP1 staining than the *Gsx2 *mutants (Figure 4D), indicating that only a rudimentary striatum is present in these brains.

The striatum is composed of two anatomically and neurochemically distinct compartments termed the patch and matrix [22]. The striatum-enriched phosphoprotein DARPP-32 has been shown to mark the forming patch compartment at perinatal time points [23] (Figure 4E). DARPP-32 is severely reduced in the *Gsx2 *mutant striatum (Figure 4F) [1,2,5,24] while its expression was only moderately reduced in the *Ascl1 *mutants (Figure 4G). Interestingly, no DARPP-32-positive neurons were observed in the *Gsx2;Ascl1 *double mutant striatum (Figure 4H), a finding that is identical to that previously observed in the *Gsx1;Gsx2 *double mutant striatum [5]. Calbindin is known to mark the matrix compartment in the mature striatum [22]. As previously reported [5], calbindin expression is increased in the forming *Gsx2 *mutant striatum (Figure 4J) while a clear reduction in its expression was seen in the *Ascl1 *mutant striatum (Figure 4K). The rudimentary striatum present in the *Gsx2;Ascl1 *double mutant striatum did express calbindin (Figure 4L). Again, this was similar to that previously observed in the *Gsx1;Gsx2 *double mutant striatum [5]. Thus, the similarities in the phenotypes observed in the *Gsx2;Ascl1 *and *Gsx1;Gsx2 *double mutants suggest that Ascl1 is required for the Gsx1-based striatal recovery in *Gsx2 *mutants.

**Figure 5:**
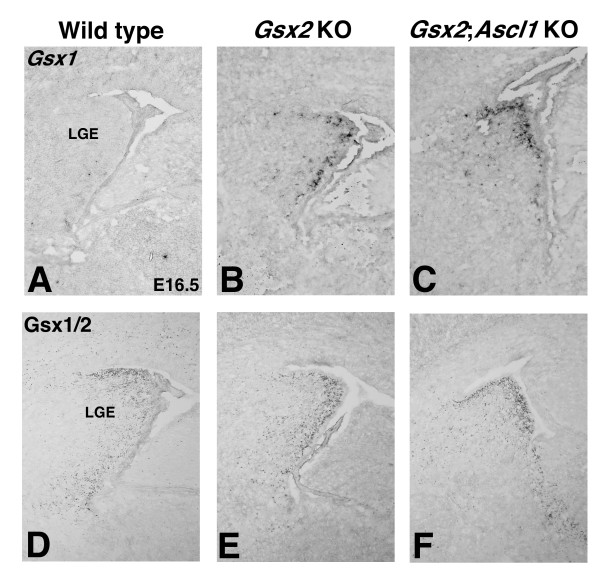
**Gsx1 expands throughout the *Gsx2;Ascl1 *mutant lateral ganglionic eminence (LGE)**. **(A-C) ***Gsx1 *expression is barely present in the E16.5 wild type LGE (A) but appears to be expressed similarly in *Gsx2 *(B) and *Gsx2;Ascl1 *mutant (C) LGEs. **(D-F) **The Gsx1/2 antibody can be used in *Gsx2 *mutants to visualize Gsx1 protein expression and not only is gene expression expanded in the mutants compared to wild type (D) but Gsx1 protein is found in a similar pattern in the *Gsx2 *mutant (E) and *Gsx2;Ascl1 *mutant (F) LGEs. The Gsx1/2 staining in the wild type mostly reflects Gsx2 expression since very little Gsx1 expression in seen in the wild type LGE (A).

In order to determine whether Ascl1 is required downstream of Gsx1 in a *Gsx2 *mutant, we examined the expression of Gsx1 in *Gsx2;Ascl1 *double mutants. If Ascl1 is required for Gsx1 to expand throughout the *Gsx2 *mutant LGE, then the similarities in the *Gsx2;Ascl1 *and *Gsx1;Gsx2 *double mutant phenotypes would be easily explained by the lack of Gsx1 in LGE progenitors. However, this is not the case, because we observed both *Gsx1 *gene expression and Gsx1/2 staining in the *Gsx2;Ascl1 *double mutant LGE (Figure 5C, F). Indeed, the level and extent of this expression was very similar to that seen in the *Gsx2 *mutant (Figure 5B, E). This allows us to conclude that Ascl1 acts downstream of Gsx1 in the *Gsx2 *mutant LGE.

### Olfactory bulb defects in *Gsx2;Ascl1 *double mutants

**Figure 6:**
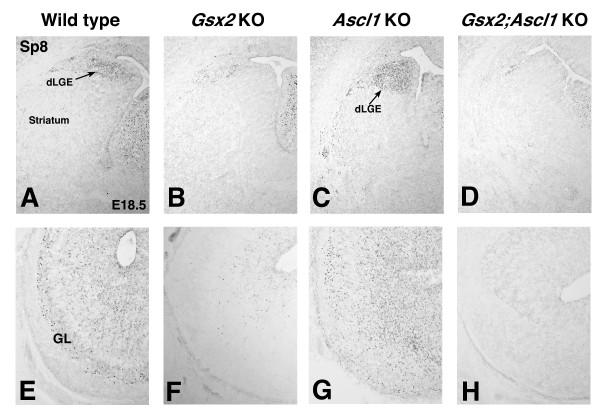
***Gsx2;Ascl1 *double mutants exhibit a more severe phenotype in the formation of the dorsal lateral ganglionic eminence (dLGE) and the generation of olfactory bulb interneurons**. **(A, B) **The Sp8 expression domain in the dLGE is reduced in *Gsx2 *mutants (B) compared to controls (A). **(D) ***Gsx2;Ascl1 *double mutants exhibit a more severe reduction in the expression of Sp8 in the dLGE (D) compared to *Gsx2 *mutants (B). **(C) ***Ascl1 *mutants maintain Sp8 expression in the dLGE and may have a slightly expanded expression domain. **(E, F) **Sp8 expressing interneurons are reduced in *Gsx2 *mutant olfactory bulb (F) compared to controls (E). **(H) ***Gsx2;Ascl1 *mutant displays nearly a complete loss of Sp8 expression in the olfactory bulb. **(G) **Sp8 expression is observed in *Ascl1 *mutant olfactory bulbs, but in a slightly disorganized pattern (compare (G) to (E)). gl, glomerular layer.

Unlike striatal neurons that are largely produced at embryonic stages, olfactory bulb interneurons are generated over a protracted period, starting around E14 and with the majority produced during the first 2 weeks after birth [25]. Since *Gsx2 *mutants die shortly after birth [4], only the olfactory bulb interneurons that are generated at embryonic stages can be assayed. Previous studies [1,3,5,6] have shown that *Gsx2 *mutants exhibit defects in the development of these neurons at birth. The olfactory bulb interneurons produced at embryonic time points have been suggested to originate, at least in part, from the dorsal (d)LGE [26]. We have recently shown that the zinc finger transcription factor Sp8 marks the dLGE as well as olfactory bulb interneurons [27] (Figure 6A, E). In *Gsx2 *mutants, the number of Sp8 expressing cells is dramatically reduced in both the dLGE and olfactory bulb (Figure 6B, F) [27]. Although *Ascl1 *mutants have been shown to have olfactory bulb interneuron defects [14,28], the expression of Sp8 in these mutants is not reduced in the dLGE at E15.5 [17]; rather, it appears as if more cells are seen in this region streaming laterally towards the ventrolateral telencephalon by E18.5 (Figure 6C). Moreover, there appear to be similar numbers of Sp8-positive cells within the *Ascl1 *mutant olfactory bulb compared to wild type, although their distribution appears somewhat disorganized (Figure 6G). Conversely, in the *Gsx2;Ascl1 *double mutants the expression of Sp8 is reduced, even when compared to the *Gsx2 *mutants (Figure 6B, D). Indeed, most sections of the double mutant olfactory bulb lack any Sp8-positive cells (Figure 6H).

**Figure 7:**
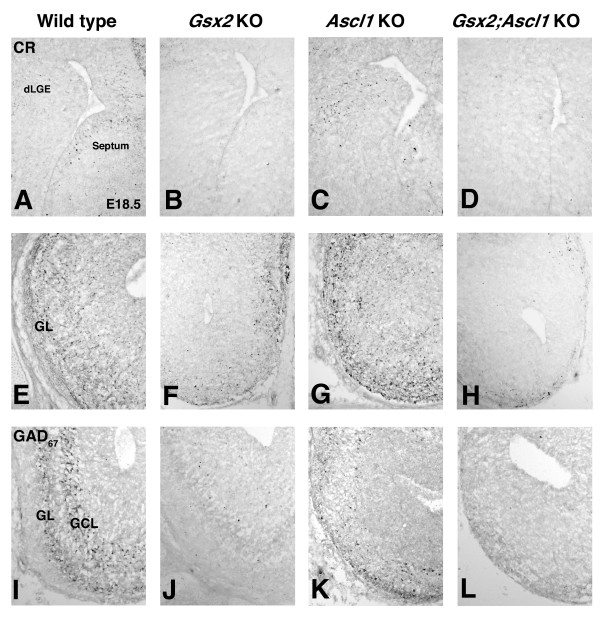
**Olfactory bulb interneuron subtype specification in *Gsx2*, *Ascl1 *and *Gsx2;Ascl1 *mutants**. **(A-H) **Calretinin (CR) staining in the dLGE (A-D) and olfactory bulb (E-H) in E18.5 wildtype (A, E), *Gsx2 *mutant (B, F), *Ascl1 *mutant (C, G) and *Gsx2;Ascl1 *mutants (D, H). Note that CR cells in the dLGE and olfactory bulb are severely depleted in *Gsx2 *and *Gsx2;Ascl1 *mutants while there appear to be similar if not more numbers of CR neurons in the *Ascl1 *mutants compared to wild type. **(J, L) **GAD_67 _(glutamic acid decarboxylase (67 kDa)) staining is severely reduced in *Gsx2 *mutants (J) and nearly absent in *Gsx2;Ascl1 *mutants (L). **(I, K) **In comparison, Ascl1 mutants (K) show a more modest reduction in GAD_67 _staining but it is still quite severe when compared to the wild-type olfactory bulb (I). GCL, granule cell layer; GL, glomerular layer.

Sp8 is required for normal development of the calretinin (CR)-expressing subtype of olfactory bulb interneurons [27]. In addition to the dLGE, the septum has also been suggested to give rise to olfactory bulb interneurons [29], and more recent results suggest that the septum may also represent a region where the CR interneurons originate [30,31]. Indeed, CR positive neurons can be seen in the wild-type dLGE and even more so in the septum at E18.5 (Figure 7A) as well as in the forming glomerular layer of the olfactory bulb (Figure 7E). As might be predicted from the Sp8 staining, the *Gsx2 *mutants showed reductions in CR interneurons (Figure 7B, F), while the *Ascl1 *mutants did not appear to exhibit reduced numbers of CR positive cells (Figure 7G) and at least in portions of the dLGE may even contain increased numbers of these cells (Figure 7C). Furthermore, the *Gsx2;Ascl1 *double mutants showed a more severe reduction in CR staining than the Gsx2 mutants (Figure 7D, H). Previous studies [1,3,5,6,14,28] have shown that *Gsx2 *(Figure 7J) and *Ascl1 *mutants (Figure 7K) exhibit reductions in glutamic acid decarboxylase (67 kDa) (GAD_67_)-positive olfactory bulb interneurons (GAD_67 _is a rate limiting enzyme in GABA production). These appear to be compounded in the *Gsx2;Ascl1 *double mutants where essentially no GAD_67_-positive cells were observed in the olfactory bulb at this time point (Figure 7L). Taken together, these data suggest that *Ascl1 *functions downstream of *Gsx2 *to regulate aspects of olfactory bulb interneuron diversity. Indeed, it appears that *Gsx2 *is required for many, if not all, of the interneuron subtypes to be properly generated, while *Ascl1 *is more crucial for the generation of GAD_67 _(that is, GABAergic) and dopaminergic interneurons [28].

### Notch signaling in *Gsx2;Ascl1 *double mutants

**Figure 8:**
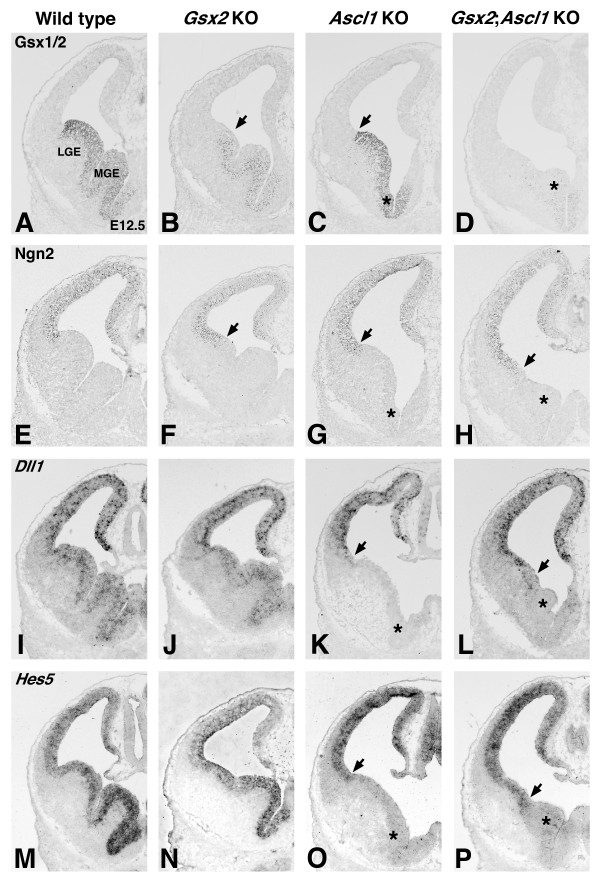
**Notch signaling in the lateral ganglionic eminence (LGE) of *Gsx2;Ascl1 *mutants is improved from that in *Ascl1 *mutants**. **(A)**_Gsx1/2 staining in the E12.5 wild type ventral telencephalon. **(B, C) **Gsx1/2 staining illustrates the expansion of Gsx1 in the *Gsx2 *mutant LGE (B) as well as the expression of Gsx proteins in the *Ascl1 *mutant LGE (C); arrows point to the dorsal limit of Gsx expression. **(D) **Note that the Gsx1 expansion is delayed in *Gsx2;Ascl1 *mutants at this stage but, as shown earlier, this recovers at later stages. **(E, F, H) **The proneural protein Ngn2 is normally expressed in pallial progenitors (E) but in the *Gsx2 *(F) and *Gsx2;Ascl1 *mutants (H) the ventral limit of Ngn2 expression (arrows in (F, H)) has expanded ventrally into the mutant LGE. **(G) **In contrast, the ventral limit of Ngn2 expression in *Ascl1 *mutants (arrow) appears to be similar to that in wild type (E). **(I-P) **The status of Notch signaling can be assessed by the expression of *Dll1 *and *Hes5*. In wild types, *Dll1 *(I) and *Hes5 *(M) are expressed in ventricular zone (VZ) progenitors along the dorsal-ventral axis of the telencephalon. As is the case in the wild types, Gsx2 mutants appear to express *Dll1 *(J) and *Hes5 *(N) throughout the telencephalic VZ, while the *Ascl1 *mutants exhibit expression only in the dorsal telencephalon (K, O) corresponding with Ngn2 expression. Although the *Gsx2;Ascl1 *mutants do not show *Dll1 *(L) and *Hes5 *(P) expression in the ventral-most telencephalon (that is, medial ganglionic eminence (MGE) remnant indicated by asterisk) these Notch effectors are expressed in the mutant LGE progenitors unlike the case in Ascl1 mutants. Asterisks in (C, D, G, H, K, L, O, P) indicate the remnant of the MGE.

Previous studies have shown that Notch signaling is required for normal LGE/striatal development [16,32]. Moreover, *Ascl1 *mutants exhibit reduced Notch signaling [14,16]. It is possible, therefore, that the phenotypes observed in the *Gsx2;Ascl1 *double mutants are a result of compound effects of a loss of Notch signaling together with distinct Gsx2 requirements. To address this, we examined the expression of factors in the Notch signaling pathway, Ngn2, Dll1 and Hes5, in relation to Gsx1/2 expression. In *Gsx2 *mutants, Ngn2 was shifted ventrally into the LGE as previously described (Figure 8F) [1-3], although it appeared to be directly abutting the ventrally shifted Gsx1/2 staining (Figure 8B). Indeed, both *Dll1 *(Figure 8J) and *Hes5 *(Figure 8N) were continuously expressed throughout the *Gsx2 *mutant LGE. In the *Ascl1 *mutants, Gsx1/2 staining was present up to the normal pallio-subpallial boundary (Figures 3A and 8C) and Ngn2 staining abutted it at its normal ventral position (compare Figure 8E and 8G). This theoretically leaves no proneural gene expression in the LGE and, in fact, both *Dll1 *(Figure 8K) and *Hes5 *(Figure 8O) staining was absent, as previously described [14]. This is not the case in *Gsx2;Ascl1 *double mutants, where Ngn2 was observed to extend ventrally into the double mutant LGE and improvement in *Dll1 *(Figure 8L) and *Hes5 *(Figure 8P) expression was observed, at least within the presumptive LGE region, compared to *Ascl1 *mutants. This indicates that the Notch signaling defects observed in *Ascl1 *mutants are, in part, due to Gsx2 expression remaining in the LGE. Thus, it appears that Ascl1 performs a non-proneural function in the Gsx1-mediated recovery observed in the *Gsx2 *mutant. Interestingly, it also seems that *Ascl1 *plays a role in the timing of the Gsx1 expansion into the *Gsx2 *mutant LGE as the *Gsx2;Ascl1 *double mutants showed much less Gsx1 (as marked by Gsx1/2 staining) expression in the presumptive LGE at E12.5 (Figure 8D) when compared to later time points (for example, E16.5; Figure 5C, F).

## Discussion

The study of knock-out mice is essentially an investigation into the compensatory mechanisms (or lack thereof) when any given gene is inactivated. In the case of Gsx2, it has previously been shown that Gsx1 is involved in the partial recovery observed in these mutants [5,6]. What remained unclear was why the Gsx1-dependent compensation was not more effective in restoring normal development. In addition to a delayed upregulation of *Gsx1 *in the *Gsx2 *mutant LGE [5,6], we provide novel data here showing that Gsx1 is expressed only in a subset of LGE cells that would normally express Gsx2. Interestingly, these cells are largely located at the boundary between the VZ and SVZ, similar in location to that of Ascl1 expressing cells. Based on the facts that the striatal phenotype of the *Gsx2;Ascl1 *mutants is nearly identical to that observed in *Gsx1;Gsx2 *mutants [5,6] and that Gsx1 expands in the *Gsx2;Ascl1 *mutants in a similar way to that observed in *Gsx2 *mutants, we conclude that *Ascl1 *is required downstream of *Gsx1 *for this recovery. These findings suggest that there are *Ascl1*-dependent and *Ascl1*-independent pathways for LGE development. This is in agreement with recent studies by Long *et al*. [17,19] showing that Dlx1/2 and Ascl1 regulate parallel and overlapping pathways in LGE specification. Furthermore, our results indicate that the *Ascl1*-dependent pathway for LGE specification appears to be independent of its well-known role in regulating the Notch signaling pathway.

The mechanism by which *Gsx1 *is upregulated in the *Gsx2 *mutant LGE has been unclear. It does not appear that Gsx2 represses Gsx1 expression because only a subset of the cells that normally express Gsx2, particularly those at the VZ/SVZ boundary, are Gsx1-positive in the *Gsx2 *mutant LGE. It seems possible, therefore, that Gsx1 can only be expressed in certain cell types or in cells that have reached a particular level of maturation (that is, cells transitioning from the VZ to the SVZ). Indeed, it appears that Gsx1-positive cells in the medial ganglionic eminence region also reside largely in the VZ/SVZ boundary region (for example, Figure 2C). Interestingly, at early stages (that is, E12.5) in the *Gsx2 *mutants, the LGE SVZ does not form, and only after Gsx1 has expanded throughout the mutant LGE (that is, by E14–15) does the it do so in this mutant [2,3,5]. Together with the current findings, these results suggest that Gsx1 may be expressed in more mature progenitors and might even play a role in the maturation process.

Ascl1 has previously been implicated in the development of the striatum and olfactory bulb interneurons [14-17,28]. In general, however, the requirement for Ascl1 in striatal and olfactory bulb development is not as great as that for Gsx2. In fact, the striatum of the *Ascl1 *mutant is only slightly reduced in size when compared to the wild type [14] (Figure 4). Moreover, the reduction in dopaminergic and GABAergic olfactory bulb interneurons [28] is not as severe as that observed in *Gsx2 *mutants [5,6]. Although striatal development is only modestly affected by the loss of *Ascl1*, we show here that the added loss of *Gsx2 *results in a nearly complete loss of striatal development. This result is identical to that previously reported for *Gsx1;Gsx2 *double mutants [5,6]. Thus, *Ascl1 *is absolutely essential for the *Gsx1*-mediated recovery observed in *Gsx2 *mutants. While Ascl1 appears to be downstream of Gsx2 [1-3], the relationship between Gsx1 and Ascl1 appears to be more complex. The loss of Gsx1 and Gsx2 severely depletes the expression of Ascl1 throughout embryogenesis [5,6], suggesting that both are genetically upstream; however, our findings here also demonstrate a delay in the expression of Gsx1 in *Gsx2;Ascl1 *double mutants at early stages (for example, Figure 8D), potentially implicating Ascl1 in feedback regulation of Gsx1 expression.

Ascl1 is a known regulator of the Notch signaling pathway [14,16] and Notch signaling has previously been implicated in controlling striatal development [16,32]. It does not seem that the striatal defects observed in the *Gsx2;Ascl1 *double mutants, described here, are simply due to compound effects of the loss of Gsx2 and impaired Notch signaling because we observed an improvement in Notch signaling (as indicated by *Hes5 *and *Dll1 *expression) within LGE progenitors of the *Gsx2;Ascl1 *double mutants compared to *Ascl1 *mutants. Our interpretation of this result is that *Gsx2;Ascl1 *mutants are similar to *Gsx2 *mutants in that Ngn2 is allowed to expand ventrally into the LGE and, as a result, Notch signaling is improved. Clearly, Ascl1 plays a role in regulating Notch signaling within LGE progenitors [14,16]; however, the fact that striatal development is not more severely affected in the *Ascl1 *mutant could suggest that Gsx2 normally works through another gene encoding a basic helix-loop-helix (bHLH) factor to regulate aspects of LGE neurogenesis.

A somewhat surprising finding that we observed in the *Ascl1 *mutants was that Gsx2 expression appeared to be increased at perinatal stages. This is not the case at early time points (for example, E12.5) and suggests that Ascl1 may play a role in depleting the Gsx2 progenitors during embryogenesis. The increased Gsx2 in the *Ascl1 *mutant LGE correlated well with the expression of Sp8, a zinc finger transcription factor that has previously been shown to be dependent on Gsx2 expression [27].

Previous studies have described a reduction in dopaminergic and GABAergic interneurons in the *Ascl1 *mutants [14,28]; however, no data on other subtypes have been provided. We show here that unlike the dopaminergic and GABAergic subtypes, the CR interneurons are not reduced and may, in fact, be increased. The neurotransmitter of this subtype remains somewhat unclear. Recent reports suggests that as few as 14% are GABAergic [33], while others suggest that most if not all are GABAergic [30,34]. Our data seem to support the former possibility (at least at this stage of development) since the reduction in GABAergic neurons (as marked by GAD_67_) is not paralleled by CR-positive cells in the *Ascl1 *mutant olfactory bulb. We have recently shown that the zinc finger transcription factor Sp8 is required for the normal development of the CR interneurons in the olfactory bulb [27]. Accordingly, we found that in *Ascl1 *mutants at late stages of development, Sp8 staining is maintained in the dLGE and olfactory bulbs. Because Gsx2 is required for Sp8 expression in the dLGE and the latter is essential for normal CR interneuron production, it seems likely that the sequential expression of these two transcription factors cooperate to generate this interneuron subtype. However, despite that Gsx2 appears to function upstream of Ascl1, this bHLH factor does not actively promote CR interneuron development.

The origin of distinct subtypes of olfactory bulb interneurons has recently been the subject of considerable attention. Kohwi *et al*. [30] have recently suggested that CR interneurons arise from pallial and septal regions but not the dLGE. On the other hand, De Marchis *et al*. [35] found that these interneurons were generated from the postnatal region of the SVZ that directly derives from the dLGE. In support of this, Merkle *et al*. [31] showed that at least some CR neurons are derived from the rostral dorsal SVZ (a likely derivative of the dLGE). Although CR interneurons start to be produced at embryonic time points, a recent study by Batista-Brito *et al*. [36] have shown that most are generated at postnatal time points. Our data show that at least a few CR neurons are present in the late embryonic dLGE and that *Ascl1 *mutants appear to exhibit enhanced CR neuron production in the dLGE and possibly olfactory bulb. Thus, Ascl1 may play a role in the temporal regulation of CR interneuron production from the dLGE and its SVZ derivatives. In any case, our findings clearly demonstrate that the dLGE is a significant source of CR interneurons that are generated at embryonic time points; however, we cannot exclude the contribution of the septum in the generation of these interneurons as shown by Merkle *et al*. [31], particularly at early postnatal stages. Indeed, Gsx2 is expressed at high levels in both the dLGE as well as in the dorsal portion of the septum (Figure 2) and CR staining in the septal region is also lost in the Gsx2 mutant (Figure 7). Regardless of their origin, it seems that all subtypes of olfactory bulb interneurons, at least at embryonic time points, require Gsx2 for their normal production.

## Conclusion

Our data show that Gsx1 compensates for the loss of *Gsx2 *gene function in only a subpopulation of the LGE progenitors that normally express Gsx2, which may explain why the compensation is not more complete. Additionally, we show that Ascl1 is an obligate factor for Gsx1 in the recovery process and that this is independent of its well-known proneural function.

## Methods

*Gsx2 *[4] and *Ascl1 *[37] mice were genotyped as previously described [2,14]. Interbreeding between *Gsx2 *and *Ascl1 *heterozygotes was performed to generate *Ascl1;Gsx2 *double heterozygotes, which were subsequently crossed to generate *Ascl1;Gsx2 *double homozygous mutants.

**Figure 9:**
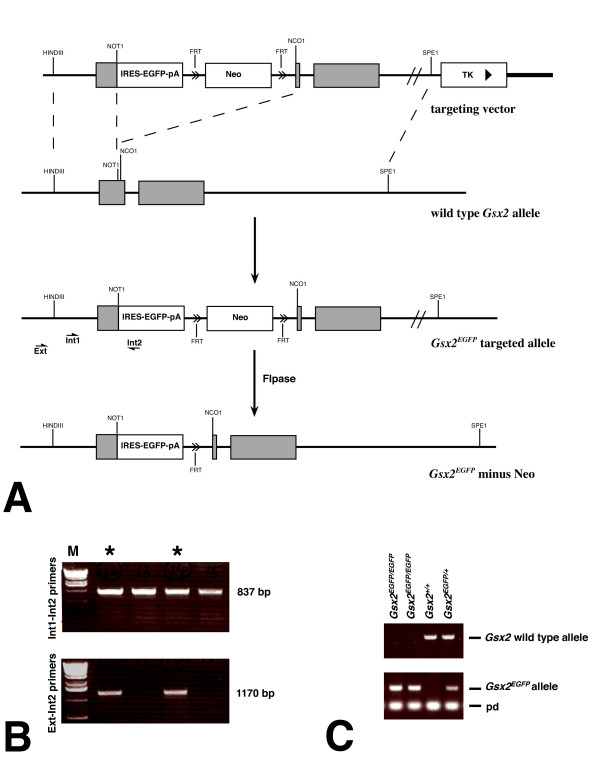
**Targeting scheme to generate the *Gsx2*^*EGFP *^knock-in allele**. **(A) **Using homologous recombination in embryonic stem cells an IRES-enhanced green fluorescent protein (EGFP) cassette was inserted in the first exon of the Gsx2 between an *Nco*1 and *Not*1 site. This removed 125 bp of the coding region of the first exon but left the exon-intron structure intact. The polyA signal at the end of the IRES-EGFP cassette effectively terminated the message and, as shown in Figure 1, no Gsx2 protein is observed when the *Gsx2*^*EGFP *^allele is bred to homozygosity. The Neomycin (Neo) cassette was removed by breeding the mice with β-actin-Flpase mice [38]. These *Gsx2*^*EGFP *^minus Neo mice were exclusively used in this study. **(B) **Correctly targeted embryonic stem cells were identified using the primers indicated as half arrows in (A). **(C) **Embryos are genotyped using primers to detect the *Gsx2*^*EGFP *^allele and using *Gsx2 *primers that include one sequence in the deleted region of the first exon. M, DNA marker; pd, primer dimmer.

*Gsx2*^*EGFP *^knock-in mice were generated by inserting an IRES-EGFP-pA cassette (Clonetech, Mountain View, CA, USA) into the first exon of *Gsx2 *between the *Not*I and *Nco*I sites (Figure 9A). Specifically, a 9-kb genomic fragment encompassing the *Gsx2 *locus was isolated by a *Hin*dIII digest of a *Gsx2*-positive 129 BAC and subcloned into the *Hin*dIII site of pBluescript SK (Stratagene, La Jolla, CA, USA). The targeting vector backbone was previously described in Bell *et al*. [38]. A 6.4-kb *Nco*1/*Spe*1 fragment from the 9-kb *Gsx2 *genomic region was blunted and subcloned into the *Hpa*1 site of cre/lox targeting vector to be used as the 3' homology arm. A 1-kb *Hin*dIII/*Not*1 fragment from the 9-kb *Gsx2 *genomic region was blunted and subcloned into the *Sma*1 site of pBluescript SK (called 5'arm-PBS). The pIRES2-EGFP vector (Clonetech) was digested with *Afl*II, blunted, and redigested with *Nhe*1 to release IRES-EGFP. IRES-EGFP was cloned into 5'arm-PBS, which was digested with *Sal*1, blunted, and redigested with *Spe*1 (5'arm-EGFP-PBS). The 5'arm-IRES-EGFP was released with a *Xho*1 digest that was blunted and subcloned into the *Pme*1 site of the cre/lox targeting vector. The *Gsx2*^*EGFP *^vector was linearized with *Sal*1 and electroporated in W4 embryonic stem (ES) cells (reviewed in [39]) and selected with G418 and gancyclovir. Correctly targeted cells were identified by PCR (Figure 9A, B) using the following primer pairs to generate products specific for the correctly targeted *Gsx2*^*EGFP*/+ ^allele: internal primer 1 (5'-cctccgcttctgttgtgact-3') with internal primer 2 (5'-cctaggaatgctcgtcaagaag-3'), which gave an 837 bp product, and an external primer (5'-cctccactacaaggccacatac3') with internal primer 2, which generated a 1,170 bp product, specific for the correctly targeted *Gsx2*^*EGFP*/+ ^allele. Two different targeted ES cell lines were used for blastocyst injection by the Cincinnati Children's Hospital Medical Center transgenic facility. Germline transmission was tested by crossing the chimeras with C57/B6 mice to obtain agouti offspring. F1 *Gsx2*^*EGFP*/+ ^mice were bred to *β-actin-FLPe *(enhanced Flpase) mice [40] obtained from Jackson Laboratory, Bar Harbor, ME, which resulted in the Neomyocin cassette flanked by FLP recombinase target (FRT) sites to be removed (Figure 9A). Embryos derived from *Gsx2*^*EGFP*/+^crosses were genotyped with the following primers: internal 2 (5'-cctaggaatgctcgtcaagaag-3') with Gsx2 int5A (5'-catcaccatcaccagcccc-3'), which generated a 225 bp product specific for the knock-in allele; and Gsx2-Int5B (5'-ccacggagattccactgcc 3') with Gsx2-1437 (5'-gcatccaccccaaatctcagtc-3'), which generated a 298 bp product specific for the Gsx2 wild-type allele (Figure 9C). The Gsx2-Int5b primer binds in the deleted region of exon 1 before the *Nco*1 site so homozygous mutants *Gsx2*^*EGFP*/*EGFP *^do not have a wild-type band.

For staging of embryos, the morning of vaginal plug detection was designated as E0.5. At least three embryos of each genotype were examined for every stage studied and marker used. Embryos were fixed overnight in 4% paraformaldehyde at 4°C, rinsed extensively in phosphate-buffered saline and cryoprotected in 30% sucrose before sectioning at 12–14 μm on a cryostat. Sections were thaw-mounted onto SuperFrost^®^/Plus slides (Fisher Scientific, Pittsburgh, PA, USA) and stored at -20°C until used.

For immunohistochemistry, primary antibodies were used at the following concentrations: rabbit anti-Ascl1 (Mash1; 1:1,000; provided by J Johnson); rabbit anti-calbindin (1:2,500; provided by P Emson); goat anti-calretinin (1:2,000; Millipore, Billerica, MA, USA); rabbit anti-Dll (pan DLX; 1:400; provided by J Khotz); rabbit anti-FoxP1 (1:4,000; provided by E Morissey); rabbit anit-GAD_67 _(1:1,000; Millipore); goat anti-GFP (1:5,000; Abcam, Cambridge, MA, USA); rabbit anti-Gsx2 (1:5,000; [2]); rabbit anti-Gsx1/2 (1:2,000; provided by M Goulding); rabbit anti-Ki67 (1:1,000; Novocastra, Newcastle, UK); rabbit anti-Ngn2 (1:1,000; provided by M Nakafuku); rabbit anti-Sp8 (1:500; [26]). The secondary antibodies for brightfield staining were biotinylated swine anti-rabbit antibodies (1:200; DAKO, Glostrup, Denmark) and biotinylated horse anti-goat antibodies (1:200; Vector Laboratories, Burlingame, CA, USA). For visualization, the ABC kit (Vector Laboratories) followed by diaminobenzidine (DAB; Sigma, St. Louis, MO, USA) as the final chromogen were utilized. The secondary antibodies for fluorescent staining were donkey anti-goat antibodies conjugated to Cy2 (Jackson Immunoresearch, West Grove, PA, USA), and donkey anti-rabbit antibodies conjugated to Cy3 (Jackson Immunoresearch).

*In situ *hybrization histochemistry was performed using digoxygenin-labeled cRNA probes as described in Toresson *et al*. [41]. Probes used were *Gsx1 *[5], *Hes5 *and *Dll1 *[14].

## Abbreviations

bHLH: basic helix-loop-helix; CR: calretinin; E: embryonic day; ES: embryonic stem; dLGE: dorsal lateral ganglionic eminence; EGFP: enhanced green fluorescent protein; GAD_67_: glutamic acid decarboxylase (67 kDa); LGE: lateral ganglionic eminence; SVZ: subventricular zone; VZ: ventricular zone.

## Competing interests

The authors declare that they have no competing interests.

## Authors' contributions

BW generated the *Gsx2*^*EGFP *^mice and carried out most of the experiments. RRW helped generate and characterize the *Gsx2*^*EGFP *^mice. ZJA helped with some of the immunohistochemistry experiments. FG provided the *Ascl1 *mice and helped conceive the experiments. KC supervised the studies and wrote the manuscript. All authors read and commented on the manuscript.
